# Evaluation of Dried Blood Spot Testing for SARS-CoV-2 Serology Using a Quantitative Commercial Assay

**DOI:** 10.3390/v13060962

**Published:** 2021-05-22

**Authors:** Davor Brinc, Mia J. Biondi, Daniel Li, Heng Sun, Camelia Capraru, David Smookler, Muhammad Atif Zahoor, Julia Casey, Vathany Kulasingam, Jordan J. Feld

**Affiliations:** 1 Department of Laboratory Medicine and Pathobiology, University of Toronto, Toronto, ON M5S 1A8, Canada; davor.brinc@uhn.ca (D.B.); vathany.kulasingam@uhn.ca (V.K.); 2 Department of Clinical Biochemistry, University Health Network, Toronto, ON M5G 2C4, Canada; heng.sun@uhn.ca; 3 Viral Hepatitis Care Network (VIRCAN) Study Group, Toronto Centre for Liver Disease, Toronto, ON M5G 2C, Canada; mia.biondi@mail.mcgill.ca (M.J.B.); camelia.capraru@uhnresearch.ca (C.C.); david@vircan.ca (D.S.); atif.zahoor@uhnresearch.ca (M.A.Z.); julia.casey@mail.utoronto.ca (J.C.); 4 Institute of Medical Science, University of Toronto, Toronto, ON M5S 1A8, Canada; daniell.li@mail.utoronto.ca

**Keywords:** SARS-CoV-2, COVID-19, dried blood spot, serology, antibody

## Abstract

Dried blood spots (DBS) are commonly used for serologic testing for viruses and provide an alternative collection method when phlebotomy and/or conventional laboratory testing are not readily available. DBS collection could be used to facilitate widespread testing for SARS-CoV-2 antibodies to document past infection, vaccination, and potentially immunity. We investigated the characteristics of Roche’s Anti-SARS-CoV-2 (S) assay, a quantitative commercial assay for antibodies against the spike glycoprotein. Antibody levels were reduced relative to plasma following elution from DBS. Quantitative results from DBS samples were highly correlated with values from plasma (*r*^2^ = 0.98), allowing for extrapolation using DBS results to accurately estimate plasma antibody levels. High concordance between plasma and fingerpick DBS was observed in PCR-confirmed COVID-19 patients tested 90 days or more after the diagnosis (45/46 matched; 1/46 mismatched plasma vs. DBS). The assessment of antibody responses to SARS-CoV-2 using DBS may be feasible using a quantitative anti-S assay, although false negatives may rarely occur in those with very low antibody levels.

## 1. Introduction

To date, the COVID-19 pandemic has resulted in more than 3 million deaths worldwide (https://coronavirus.jhu.edu/map.html, accessed on 19 May 2021). While physical distancing and stay-at-home measures play an important role, detection and monitoring of cases is crucial to the management of the pandemic. SARS-CoV-2 serology is useful for seroprevalence studies, identifying cases of negative/indeterminate molecular results despite high clinical suspicion of COVID-19, and diagnostic assessment for multi-system inflammatory syndrome in children [1]. In addition, plasma antibody levels may determine natural and/or vaccine-derived immunity to SARS-CoV-2 infection. 

The development of serologic testing platforms has been rapid. This has included platforms for unique antibodies with combined isotypes against different viral targets, with qualitative and, more recently, quantitative platforms. However, sample collection for SARS-CoV-2 serological testing requires phlebotomy from a trained provider with access to sample processing and a central laboratory.

The use of dried blood spots (DBS), a collection method whereby capillary blood is collected onto filter paper, may facilitate more widespread testing by overcoming some of these obstacles [2,3]. Serological testing using DBS has already been validated and optimized for other viral infections including HIV and hepatitis B and C [4,5,6,7,8,9,10,11,12,13,14,15]. Compared to venous blood collection, DBS sample collection is less complex and offers the opportunity for peer or even self-collection, facilitating collection in rural and remote settings, as well as in populations who may experience geographic or structural barriers seeking healthcare in traditional settings. Collection kits could theoretically be mailed to patients who are then able to reliably collect their own samples and send them back to a testing facility [16]. This eliminates the need for person-to-person contact and healthcare visits; facilitates repetitive sampling and widespread surveillance; and allows for testing to reach populations that lack adequate testing resources, such as in rural areas and parts of low- and middle-income countries. 

Since the beginning of the pandemic, several manufacturers have developed commercial high throughput assays for SARS-CoV-2 serology, as not all laboratories have the capacity to develop in-house assays. Commercial assays also allow for comparisons between labs, which is important as the medical and scientific community incorporate serology testing into various aspects of clinical care. These assays have demonstrated high specificity and sensitivity using serum/plasma. Although there have been preliminary studies evaluating DBS collection for anti-SARS-CoV-2 immunoassays, DBS serology tests have yet to be used on a widespread scale [2,17,18,19,20,21,22,23,24,25,26,27,28,29,30,31,32]. Importantly, the majority of platforms evaluated have been for qualitative assays for antibodies against SARS-CoV-2, a potential gap should a quantitative option prove beneficial. 

Here, we evaluated a commercial anti-SARS-CoV-2 S Total assay (Roche) using DBS prepared from plasma-spiked RBC. Following the characterization of the assay, we then completed a head-to-head real-world comparison of matched plasma and DBS collected by finger prick from individuals with a PCR-confirmed positive history of COVID-19. 

## 2. Materials and Methods

### 2.1. Specimens

Residual plasma (Li-Heparin) samples from PCR-confirmed positive SARS-CoV-2 patients (*n* = 24, plasma collected 5–39 days after swab collection for PCR testing) and COVID-19-negative patients (*n* = 21, pre-COVID-19 plasma samples or samples from PCR-confirmed negative patient) were used for spiking red blood cells (Table S1). To prepare spiked blood samples, we mixed plasma with saline-washed (3×) O-negative red blood cells (1:1 ratio). A total of 50 µL of prepared whole blood was then added to completely fill 5 spots on the Whatman 903 Protein Saver cards (GE Healthcare, Chicago, IL, USA). The approach of spiking blood samples has been used previously in the validation of COVID-19 and non-COVID-19 DBS serology testing [14,31]. Filter cards were dried for 2–3 h at room temperature in the absence of any external source of heat and processed the same day. 

Matched plasma and dried blood spots were also collected from 52 PCR-positive confirmed patients, 90 days or greater from diagnosis, and 11 healthy controls (Table S2). These 52 patients were recruited from a phase II randomized clinical trial of COVID-19 treatment [33]. Blood was collected by finger prick to fill the spots on a Whatman 903 Protein Saver card. All specimens were dried and stored at −80 °C until use. The use of residual samples was exempt from quality improvement (QI) review, and the use of both sets of patient samples was approved by Clinical Trials Ontario Research Ethics Board (REB) (Toronto, ON, Canada) on 9 September 2020 (CTO 2148). 

### 2.2. Dried Blood Spots

DBS processing was modified from a previously published protocol [34,35]. Two punches per spot on the filter card, with a total of 10 punches per filter card, were prepared. Each punch size had a diameter of 6.35 mm, corresponding to an area of 31.65 mm^2^ (24.99% of each filter card circle area). Punched spots were placed in 750 µL of phosphate-buffered saline (plus 0.05% Tween 20 (*v/v*) and 0.08% sodium azide (*w/v*)) in 12-well plates (Thermo Scientific BioLite Multidishes and Microwell Plate, Thermo Fisher Scientific, Waltham, MA, USA) and incubated overnight on a shaker at room temperature. Eluates were then transferred to microcentrifuge tubes, centrifuged for 2 min at 10,500× *g*, and stored at −80 °C until use. Assuming 50 µL whole blood is applied and 45% hematocrit, we had an expected dilution of plasma after DBS elution of ×11.

### 2.3. SARS-CoV-2 Antibody Assay

The quantitative Roche Elecsys Anti-SARS-CoV-2 (S) assay measuring antibodies to spike (S) glycoprotein was chosen to evaluate the relationship between serum/plasma SARS-CoV-2 antibody testing and testing from DBS. The assay detects antibodies to RBD of S glycoprotein. Threshold for positivity is greater than or equal to 0.8 U/mL. Analytical measurement range is 0.4 to 250 U/mL, with dilution 0.4 to 2500 U/mL. Imprecision at 9.06 U/mL was 1.26% on the basis of quality control material. The assay was carried out according to the manufacturers’ instructions.

### 2.4. Data Analysis

The correlation between plasma and DBS quantitative results was determined using the Pearson correlation coefficient. All statistical analysis and graphing were performed using R (https://www.R-project.org, accessed on 19 May 2021) with the following packages: tidyverse, xlsx, ggpmisc, multcomp, irr, pROC, and epiR [36]. 

## 3. Results

### 3.1. Initial Validation of Quantitative Roche S Assay Using Prepared DBS Specimens

DBS were processed by removing two punches per spot on the filter card, with a total of 10 punches per filter card, into 750 µL of PBS overnight. The optimal procedure was chosen on the basis of the magnitude of decrease of the signal observed, linear correlation between plasma and DBS, and sufficient extraction volume for multiple testing, as well as ease of processing. 

To determine the ability of commercial automated serology assay to detect antibody response to SARS-CoV-2 in DBS eluants, we mixed plasma samples from COVID-19-positive patients with a wide range of antibody titers (Table S1) with RBCs and applied them to filter cards. The samples were deliberately chosen to include both extremes of high and low signal samples to test the performance of DBS. The antibody signal obtained from eluants was compared to results from corresponding plasma samples. 

**Figure 1:**
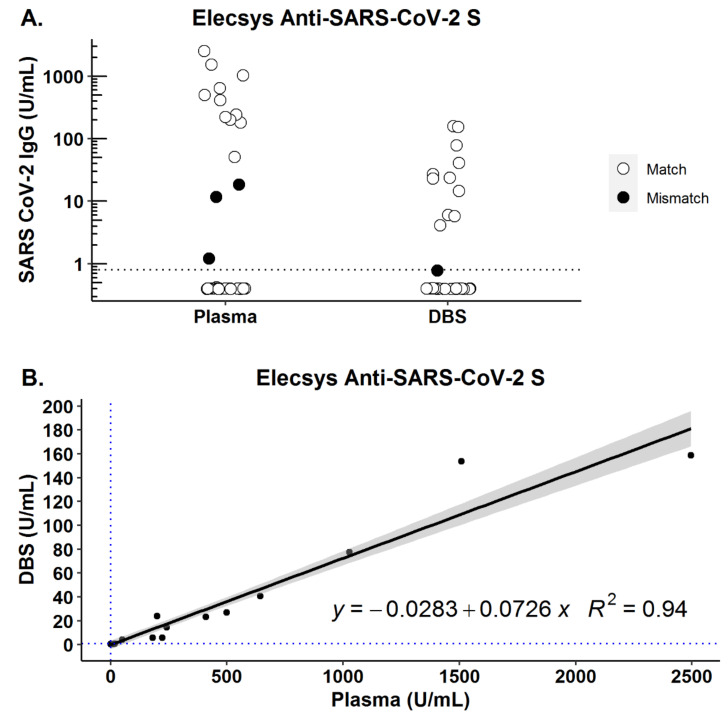
RBC mixed with plasma (1:1) were applied to filter cards, dried, and eluted. The sera and corresponding eluants were analyzed using Roche Elecsys Anti-SARS-CoV-2 (S) assay (plasma used for spiking RBC: *n* = 24 COVID-19-positive (*n* = 23 serology positive) patients; *n* = 21 COVID-19-negative (*n* = 22 serology negative) patients). The horizontal line indicates assay threshold as quoted by manufacturer for plasma/serum. (**A**) Distribution of values of DBS samples prepared from spiked RBC and corresponding plasma used for spiking for mismatched samples (●) and matched samples (ο). (**B**) Dataset from (**A**) showing correlation between concentrations obtained from plasma vs. DBS.

For all samples, the recommended threshold for detection in plasma/serum was used for both the plasma and DBS samples. A decrease in signal was observed after DBS elution; however, the correlation between plasma and DBS concentration was high (*r*^2^ = 0.94). There were no false positive DBS results, but 3/23 samples were positive in plasma but negative in DBS (Figure 1).

### 3.2. Performance of Quantitative Roche S Assay in Matched Plasma/DBS Fingerprick Collection

**Figure 2:**
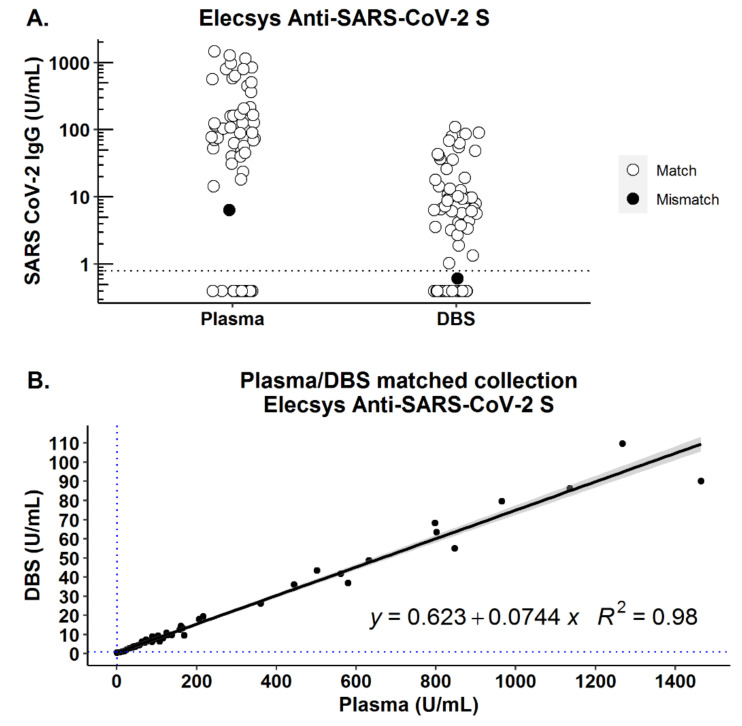
Plasma and dried blood spot collection comparison. Plasma and DBS samples collected in parallel from the same participant were processed and analyzed by Roche Elecsys Anti-SARS-CoV-2 (S) assay (*n* = 52 COVID-19-positive, *n* = 11 COVID-19-negative). Horizontal line indicates assay threshold as quoted by manufacturer for plasma/serum. (**A**) Distribution of values in plasma and DBS samples for mismatched samples (●) and matched samples (ο). (**B**) Dataset from (**A**) showing correlation between concentrations obtained from plasma vs. DBS.

Plasma and DBS were collected from 52 patients 90 days or more after their initial positive PCR positive test (mean: 136, min: 90, max: 208 days; Table S2). Of the 46 patients with positive serology from plasma, 45 (97.8%) were detected by DBS. All 17 (6 patients and 11 negative controls) samples that tested negative for SARS-CoV-2 antibodies in plasma were also negative on the corresponding DBS sample (Figure 2A, Table 1). Using the quantitative Roche assay, we found that the correlation between quantitative results obtained on plasma and DBS was very high (*r*^2^ = 0.98, Figure 2B). Clinical sensitivity and specificity were not significantly different from plasma-based performance in this group of patients. DBS (using serum/plasma-based threshold) showed 98% (95% CI: 88–100%) sensitivity and 100% (95% CI: 80–100%) specificity relative to plasma (Table 1). Of note, not all individuals who were PCR-positive had persistent antibodies by plasma on day 90 (*n* = 6); these individuals were also negative by DBS (Table 1).

## 4. Discussion

In this study, our aim was to determine the feasibility of using the commercial quantitative SARS-CoV-2 anti-S Roche assay, with no changes to assay configuration or threshold, in order to measure antibody levels from DBS specimens. We demonstrated that antibody measurement after DBS collection showed high concordance, both qualitatively and quantitatively, with standard plasma measurements, even though the antibody levels are predictably decreased on DBS.

We used plasma spiked with RBCs to mimic whole blood that is normally collected on DBS for initial assay optimization and comparisons; however, we found that the plasma-spiked DBS cards performed near identically to contemporaneously collected DBS and plasma samples, giving us confidence in the results observed (Figure S1). We found detectable antibodies in the plasma in 88% of people at least 3 months post-PCR positivity. The clinical performance of the different commercial assays, such as Abbott SARS-CoV-2 (N) IgG, DiaSorin SARS-CoV-2 S1/S2 IgG, and Roche Elecsys Anti-SARS-CoV-2 (N), have been investigated, and sensitivity greater than 80% and specificity greater than 95% 14 weeks after confirmed infection have been reported in hospitalized patients. Slightly lower sensitivity for antibodies has been observed in outpatient settings [37,38,39,40,41,42,43,44,45,46]. Roche Elecsys Anti-SARS-CoV-2 (S) is a quantitative assay that detects antibodies against the receptor-binding domain (RBD) with reported sensitivity of 84.0% 15–30 days post-PCR positivity, with no cross-reactivity leading to 100% specificity [47]. Compared to plasma, we found that antibodies detected from DBS using the Roche (S) assay performed well with a sensitivity of 98% and specificity of 100%. We also observed excellent correlation between quantitative plasma and DBS antibody levels with a predictable decrease in signal, allowing for prediction of the serum signal from the DBS signal, which would be important if thresholds to define immunity are identified. The performance of the Roche (S) assay we observed is consistent with recent research highlighting its high specificity and sensitivity with serum [47,48]. 

High concordance between antibody detection from serum/plasma versus DBS has been reported in studies using ELISA-type immunoassays and lateral flow immunoassays where sample dilution is adjusted for in DBS specimens to compensate for dilution due to filter card extraction [2,17,20,24,27,28]. One group analyzed DBS samples from 80 volunteers using an in-house-developed ELISA protocol. Detecting the presence of IgG, IgA, and IgM against the SARS-CoV-2 spike protein, they achieved a sensitivity and specificity of 98.1% and 100%, respectively, relative to serum [17]. In another study with 111 (31 COVID-19-positive) samples, a modified antibody detection by agglutination-PCR (ADAP) procedure was used with concentrated DBS eluant. They were able to achieve 100% sensitivity and specificity compared to serum/plasma for antibodies against the SARS-CoV-2 S1 spike protein subunit [18]. A recent study evaluated performance of DBS using Abbott SARS-CoV-2 IgG (N) and SARS-CoV-2 IgM (S) qualitative assays, but with sample volume modification to compensate for DBS elution, and showed high concordance between plasma and DBS samples [32].

However, while useful, these studies relied on in-house assays for antibody detection that were modified to improve performance with DBS. Most clinical laboratories rely on commercially available antibody detection tools with limited ability to adjust assay parameters, and thus we felt it was important to evaluate DBS using commercially available assays in order to understand how it might be used outside of a research context. The amount of dilution from the filter cards can be theoretically predicted and normalized; however, whether this leads to predictable decrease in antibody signal depends on assay linearity. Roche Elecsys SARS-CoV-2 S assay was shown to be linear [47]. The correlation between signal predicted from serum or plasma vs. observed DBS signal was high (Figure S2). A recent study tested the Roche assay (total N assay) and showed limited performance of DBS relative to serum. The sensitivity was only 44% relative to plasma if the plasma cut-offs were used or 89% if a derived cut-off was used, about 30× higher than the reported LOD. However, the size of the area eluted and elution buffer volume likely resulted in higher dilution of sample compared to the current study. The study also observed variation in the quality of DBS collected [29]. 

Several parameters affect the amount of predicted elution from the filter cards [26,28,49]. The number of circles spotted with blood, the punch size, and the number of punches will determine the size of the surface area exposed to elution buffer. The volume of elution buffer is also important. Our initial choice of elution procedure was driven by literature review of DBS testing of non-SARS-CoV-2 serology and focused on several parameters that could affect dilution of the final eluant. We found modifications of the standard procedure had limited impact on the performance of DBS, but overall, we found that 10 spots from 5 cards eluted in 750 µL of PBS gave the most consistent results. A recent study also showed the effect of DBS size and punch location on the observed DBS signal [26]. DBS specimens have been shown to be stable at room temperature, 4 °C and −20 °C, at ambient or low humidity conditions for at least 28 days [26]. An automated DBS extraction procedure is also being developed [23]. 

Our study has limitations. The assay we report does not specifically test for neutralizing antibodies. Some studies have reported potential use of DBS collection for neutralizing antibodies as well [50]. However, it has been found that high receptor binding domain-specific antibody titers may be indicative of the presence of neutralizing antibodies against SARS-CoV-2 [51,52,53,54]. Further validation of DBS collection may be required once the level of neutralizing antibodies that confers protection has been identified. We had a limited number of samples with very low antibody levels, the situation in which the sensitivity of DBS declines and there is a greater risk for misclassification; however, the clinical importance of this scenario is questionable given that very low levels of antibody may not be protective. Quantitative assays are yet to establish traceability to higher order reference material, which would facilitate comparison between different assays and establishment of universal thresholds of immunity if possible. Recently the WHO prepared an international standard and reference panel for SARS-CoV-2 immunoglobulins, which will hopefully help in assay standardization [55]. We used the same thresholds for DBS as for plasma. New cut-offs for DBS could potentially be developed; however, with the very small difference between the limit of detection (LOD) and the thresholds for positivity (e.g., 0.4 U/mL vs. 0.8 U/mL on Roche S assay), it may be difficult to find a DBS-specific cut-off. Furthermore, changing the cut-off on the basis of expected dilution resulted in cut-off values that fell below the limit of quantitation (LOQ). With these limitations in mind, it will be important for individual laboratories to conduct their own internal validation studies, as well as to evaluate diagnostic limitations while balancing clinical utility. This would include negative and positive controls, as well as evaluating for cross-reactivity for seasonal coronaviruses. 

As SARS-CoV-2 vaccines continue to roll out, the feasibility of DBS serology testing can allow for widespread monitoring of vaccine responses. In addition, DBS opens the door to identifying unknown previous cases of COVID-19 (i.e., asymptomatic infections), which may be helpful in developing targeted vaccination strategies and understanding the overall burden and case number in the pandemic. The use of DBS for COVID-19 serology testing has been tested in neonates and in COVID-19 seroprevalence studies [22,25,30]. We show promising performance of quantitative Roche SARS-CoV-2 S assay on DBS samples, with high correlation with plasma antibody levels and concordance with serum-based positivity classification.

## Figures and Tables

**Table 1:** Clinical performance of DBS vs. plasma for PCR-confirmed COVID-19 detection.

Comparisons	Threshold (U/mL)	True Negatives	True Positives	False Negatives	False Positives	Sensitivity	Specificity
Plasma vs. PCR-confirmed COVID-19	0.8	11	46	6	0	0.88 (95% CI: 0.77, 0.96)	1 (95% CI: 0.72, 1)
DBS vs. PCR-confirmed COVID-19	0.8	11	45	7	0	0.87 (95% CI: 0.74, 0.94)	1 (95% CI: 0.72, 1)
DBS vs. plasma-positive COVID-19	0.8	17	45	1	0	0.98 (95% CI: 0.88, 1)	1 (95% CI: 0.8, 1)

## Supplementary Materials

The following are available online at https://www.mdpi.com/article/10.3390/v13060962/s1, Figure S1: Comparison between Anti-S concentration obtained from DBS prepared from capillary blood (“Direct DBS collection”) and DBS prepared from RBC spiked with corresponding plasma (“DBS from mixed plasma + RBC”). Figure S2: Correlation between antibody level measured in eluted DBS and predicted dilution of plasma. Table S1: Patient samples selected for initial DBS validation. Table S2: Patient samples selected for DBS SARS-CoV-2 serology validation using matched filter card/plasma collection, 90 days or more post-infection, and negative controls.
