# Supplementary material for: Evaluation of Dried Blood Spot Testing for SARS-CoV-2 Serology Using a Quantitative Commercial Assay
Source: Viruses. 2021 May 22;13(6):962. doi: 10.3390/v13060962 (PMC8224688; doi:10.3390/v13060962)
Supplement: Supplementary file 1 [file viruses-13-00962-s001.zip › viruses-1150288-Supplementary Materials.pdf]

Supplementary Materials

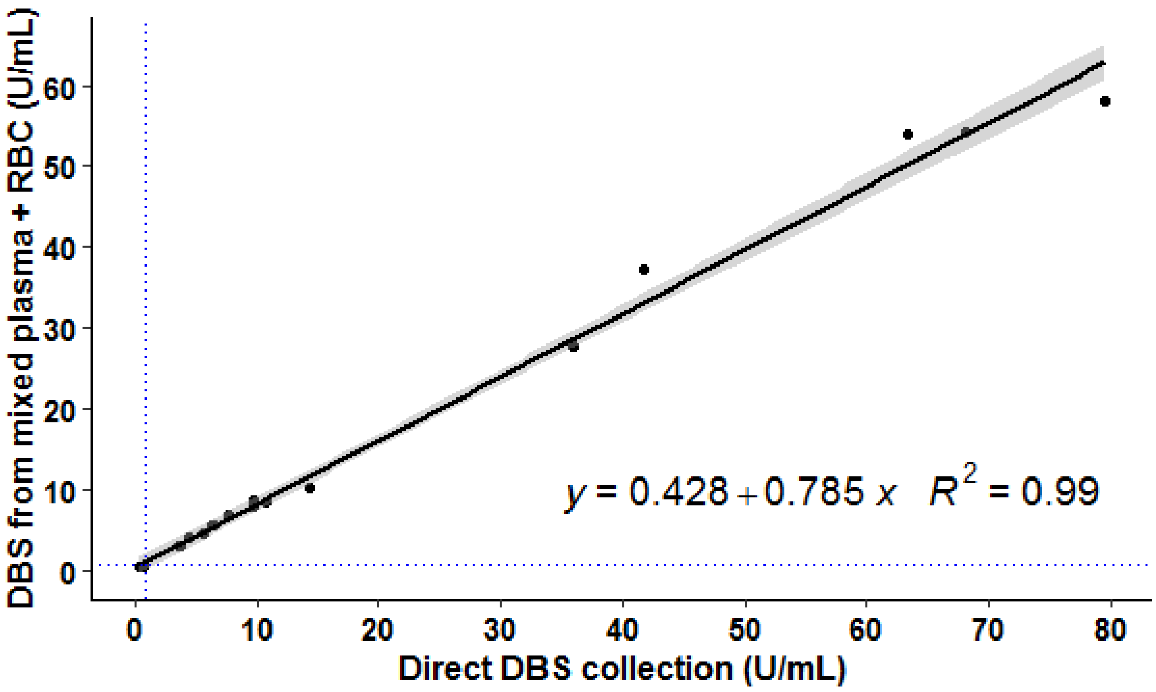

Figure S1. Comparison between Anti-S concentration obtained from DBS prepared from capillary blood ("Direct DBS collection") and DBS prepared from RBC spiked with corresponding plasma ("DBS from mixed plasma + RBC"). Plasma and corresponding fingerprick capillary blood spotted onto filter paper were collected from COVID19 patients. (n=20 COVID19-positive). Horizontal line indicates plasma/serum-based cutoff as indicated by the manufacturer. All samples were run on Roche Elecsys Anti-SARS-CoV-2 (S) assay.

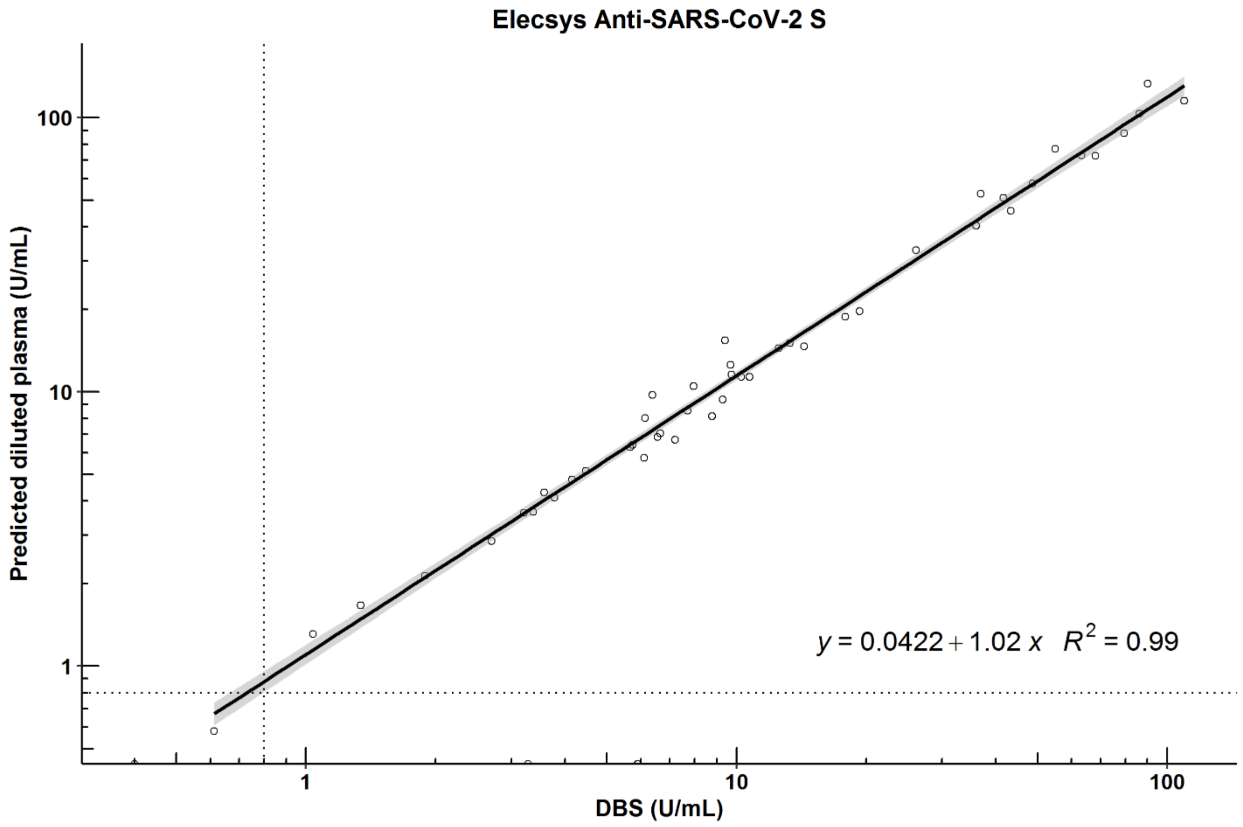

Figure S2. Correlation between antibody level measured in eluted DBS and predicted dilution of plasma. Predicted (x11) dilution of plasma antibody level using matched plasma/DBS collection sample set on Roche Elecsys Anti-SARS-CoV-2 assays (n=52 COVID19-positive, n=11 COVID19-negative). Horizontal line indicates plasma/serum-based cutoff as indicated by manufacturer.

Table S1. Patient samples selected for initial DBS validation.

**Random ID**	**PCR**	**Days since PCR-positive**	**SARS-CoV-2, Roche (S Total), Plasma (U/mL)**
Sample 9	Positive	27	NA
Sample 2	Positive	15	NA
Sample 5	Positive	39	NA
Sample 7	Positive	12	NA
Sample 8	Positive	5	NA
Sample 3	Positive	6	NA
Sample 1	Positive	17	NA
Sample 10	Positive	21	NA
Sample 6	Positive	16	NA
Sample B12	Positive	33	18.14
Sample B5	Positive	36	1028
Sample B7	Positive	27	181.1
Sample B15	Positive	18	11.58
Sample B10	Positive	16	499.5
Sample B9	Positive	35	2496
Sample B2	Positive	33	241.2
Sample B6	Positive	28	410.4
Sample B3	Positive	10	**0.417**[1]
Sample B4	Positive	19	1509
Sample B13	Positive	15	199.9
Sample B14	Positive	15	643.4
Sample B11	Positive	15	1.21
Sample B8	Positive	14	222.6
Sample B1	Positive	7	50.6
Sample 4	Negative	N/A	N/A
Sample C66	Pre-covid sample	N/A	<0.4
Sample C67	Pre-covid sample	N/A	<0.4
Sample C68	Pre-covid sample	N/A	<0.4
Sample C69	Pre-covid sample	N/A	<0.4
Sample C70	Pre-covid sample	N/A	<0.4
Sample C71	Pre-covid sample	N/A	<0.4
Sample C72	Pre-covid sample	N/A	<0.4
Sample C73	Pre-covid sample	N/A	<0.4
Sample C74	Pre-covid sample	N/A	<0.4
Sample C75	Pre-covid sample	N/A	<0.4
Sample C76	Pre-covid sample	N/A	<0.4
Sample C77	Pre-covid sample	N/A	<0.4
Sample C78	Pre-covid sample	N/A	<0.4
Sample C79	Pre-covid sample	N/A	<0.4
Sample C80	Pre-covid sample	N/A	<0.4
Sample C81	Pre-covid sample	N/A	<0.4
Sample C82	Pre-covid sample	N/A	<0.4
Sample C83	Pre-covid sample	N/A	<0.4

Sample C84	Pre-covid sample	N/A	<0.4
Sample C85	Pre-covid sample	N/A	<0.4

[1]Serology and PCR-positivity discordance.

Table S2. Patient samples selected for DBS SARS-CoV-2 serology validation using matched filter card/plasma collection, 90 days or more post-infection, and negative controls.

**RandomID**	**COVID19 Status**	**Days since PCR-positive**	**SARS-CoV-2, Roche (S Total), Plasma (U/mL)**
Sample C1	PCR-confirmed	157	847.3
Sample C2	PCR-confirmed	151	52.69
Sample C3	PCR-confirmed	152	70.5
Sample C4	PCR-confirmed	141	<0.4
Sample C5	PCR-confirmed	139	23.51
Sample C6	PCR-confirmed	149	14.42
Sample C7	PCR-confirmed	145	579.8
Sample C8	PCR-confirmed	145	1465
Sample C9	PCR-confirmed	138	<0.4
Sample C10	PCR-confirmed	90	<0.4
Sample C11	PCR-confirmed	96	<0.4
Sample C12	PCR-confirmed	100	137.8
Sample C13	PCR-confirmed	123	63.25
Sample C14	PCR-confirmed	106	94.11
Sample C15	PCR-confirmed	116	39.75
Sample C16	PCR-confirmed	122	75.38
Sample C17	PCR-confirmed	121	115.5
Sample C18	PCR-confirmed	135	124.8
Sample C19	PCR-confirmed	135	77.51
Sample C20	PCR-confirmed	119	47.24
Sample C21	PCR-confirmed	119	158.4
Sample C22	PCR-confirmed	119	561.5
Sample C23	PCR-confirmed	128	103
Sample C24	PCR-confirmed	119	107.1
Sample C25	PCR-confirmed	119	127.3
Sample C26	PCR-confirmed	133	444.5
Sample C27	PCR-confirmed	126	1136
Sample C28	PCR-confirmed	152	56.8
Sample C29	PCR-confirmed	131	31.38
Sample C30	PCR-confirmed	130	<0.4
Sample C31	PCR-confirmed	129	40.15
Sample C32	PCR-confirmed	132	801.8
Sample C33	PCR-confirmed	133	216.3
Sample C34	PCR-confirmed	137	965.5
Sample C35	PCR-confirmed	139	360.9
Sample C36	PCR-confirmed	144	161
Sample C37	PCR-confirmed	147	501.9

Sample C38	PCR-confirmed	169	632.7
Sample C39	PCR-confirmed	201	798.1
Sample C40	PCR-confirmed	171	165.5
Sample C41	PCR-confirmed	171	45.27
Sample C42	PCR-confirmed	150	6.37
Sample C43	PCR-confirmed	193	73.46
Sample C44	PCR-confirmed	177	69.21
Sample C45	PCR-confirmed	208	169.5
Sample C46	PCR-confirmed	161	<0.4
Sample C47	PCR-confirmed	121	89.72
Sample C48	PCR-confirmed	116	206.5
Sample C49	PCR-confirmed	124	124.5
Sample C50	PCR-confirmed	104	1268
Sample C51	PCR-confirmed	103	18.28
Sample C52	PCR-confirmed	101	88.42
Sample C55	Negative	NA	<0.4
Sample C56	Negative	NA	<0.4
Sample C57	Negative	NA	<0.4
Sample C58	Negative	NA	<0.4
Sample C59	Negative	NA	<0.4
Sample C60	Negative	NA	<0.4
Sample C61	Negative	NA	<0.4
Sample C62	Negative	NA	<0.4
Sample C63	Negative	NA	<0.4
Sample C64	Negative	NA	<0.4
Sample C65	Negative	NA	<0.4
